# Alteration of Peripheral Resistin and the Severity of Acute Pancreatitis: A Meta-Analysis

**DOI:** 10.3389/fmed.2022.915152

**Published:** 2022-06-13

**Authors:** Jianhua Yang, Mengyao Liu, Shu Wang, Yuanxiu Gan, Xiangyu Chen, Yang Tao, Junwei Gao

**Affiliations:** ^1^Department of Critical Care Medicine, Chongqing Emergency Medical Center, Chongqing, China; ^2^Department of Osteology, Army Medical Center of PLA, Third Military Medical University (Army Medical University), Chongqing, China; ^3^Department of Emergency, Southwest Hospital, Third Military Medical University (Army Medical University), Chongqing, China; ^4^Department of Military Cognitive Psychology, School of Psychology, Third Military Medical University (Army Medical University), Chongqing, China

**Keywords:** acute pancreatitis, resistin, serum, plasma, association

## Abstract

**Introduction:**

Resistin is a small secretory adipokine which is implicated to obesity and associated diseases. Recently, plenty of research papers have been conducted to explore the association between peripheral resistin and the severity of acute pancreatitis (AP). However, the results were controversial. In this study, we aimed to confirm the effect of peripheral resistin and the development of acute pancreatitis.

**Methods:**

A comprehensive online search was performed using the PubMed, Embase, Web of Science, CNKI, and Wanfang databases up through January 20, 2022. The retrieved records and their references were screened to identify additional studies. Data were extracted to calculate the pooled Hedges' g and its 95% CI, which were selected to assess peripheral resistin levels and the severity of acute pancreatitis. Subgroup analyses, sensitivity analyses, meta-regression, and publication bias tests were also undertaken based on obtained information.

**Results:**

A total of eleven studies with 892 acute pancreatitis patients were enrolled in the study. Peripheral resistin levels were significantly increased in severe acute pancreatitis compared with mild acute pancreatitis (Hedges' g = 2.092, 95% CI: 0.994–3.190, *P* < 0.001). Subgroup analyses based on sample types and ethnicity also showed similar results. A single study did not affect our results, which was verified by sensitivity analysis. Meta-regression analyses revealed that age, gender of the included subjects, sample size, and publication year did not moderate effects on the present results.

**Conclusion:**

In our study, peripheral resistin levels were significantly elevated in patients with severe AP compared with patients with mild AP. Abnormal resistin levels may provide us some new insights in predicting the severity of AP.

## Introduction

Acute pancreatitis (AP) is one of the most common clinical syndrome initiated by pancreatic injury from a variety of pathologic mechanisms (1). Pancreatic injury is mild and self-limiting in approximately 80% of patients with AP that would find their health after brief hospitalization. Unfortunately, the other 20% of patients with AP would develop lethal severe acute pancreatitis (SAP) that requires intensive care (2, 3). Despite improvements in treatment measurements and supportive care, SAP remains one of the important causes of death during the past decade (4). Therefore, identification of patients with SAP from patients with AP is urgently needed to help determine therapeutic interventions as early as possible.

Obesity is a chronic, systemic low-grade inflammatory state characterized by high levels of peripheral proinflammatory cytokines. Currently, about 40% of adults are overweight, and obesity has now become an important health problem (5, 6). A number of recent studies have suggested that obesity is associated with the risk of severe acute pancreatitis (7, 8). It is common knowledge that abnormal release of destructive digestive enzymes from pancreatic acinar cells and systemic inflammatory response play critical roles in the development of AP (9). Pancreatic microcirculation is poorer in obese patients that are more prone to infection and SAP (10). The main problem in managing acute pancreatitis is the lack of availability of convenient indicators or scoring systems for predicting severity and necrosis in the first hours of the disease (11). Lots of animal studies have shown that obesity-related cytokines, such as leptin and adiponectin, contributed to the development of AP (12, 13). Multiple clinical trials have found these abnormal cytokine profiles in patients with AP (14, 15). It seems that adipokines are associated with acute pancreatitis.

Resistin, coded by the RETN gene, belongs to the family of resistin-like molecules. It is mainly produced by adipose tissue, inflammatory cells, and functions as a pro-inflammatory adipokine (16, 17). There are many studies reported resistin plays a major role in a series of inflammation-related diseases, such as atherosclerosis, sepsis, and cancer (18–20). Animal studies have revealed that resistin could enhance intracellular Ca^2+^ levels, NADPH oxidase activity, intracellular reactive oxygen species (ROS) production, NF-κB activity, and IL-6 expression in cerulein-stimulated AR42J cells, which may contribute to the development of AP (21). Peripheral resistin was initially thought to be a promising biomarker, which could predict severe acute pancreatitis in the early phase (22). So far, many clinical studies have tried to determine whether the levels of peripheral resistin are related to the severity of AP. However, the results were inconsistent. Thus, we conducted a meta-analysis to further investigate the effect of peripheral resistin on the severity of AP based on larger sample size.

## Materials and Methods

### Search Strategy

A systematic literature search was performed for the relevant literature in five databases (PubMed, Embase, Web of Science, China National Knowledge Infrastructure (CNKI), and Wanfang databases) up through January 20, 2022 according to the guidelines of Preferred Reporting Items for Systematic Reviews and Meta-Analysis (PRISMA) statement. The retrieval key search terms were included the following: “acute pancreatitis,” “pancreatic necrosis,” or “peripancreatic necrosis”; and “resistin.” To obtain all eligible studies, all retrieved articles and their references were checked.

### Inclusion and Exclusion Criteria

The records retrieved were screened according to the inclusion and exclusion criteria. In our meta-analysis, the inclusion criteria for the studies were as follows: (1) clinical studies evaluating the levels of resistin in severe and mild acute pancreatitis. (2) resistin detected in plasma or serum. (3) a mean and standard deviation, or sample size and *P*-value were presented in records. The exclusion criteria for the studies were as follows: (1) studies with insufficient information such as concentration or *P-value*. (2) animal study, review, comment or meeting abstract. (3) *in vitro* studies. When the retrieved studies contained overlapping data, only the data of study with the largest sample size was extracted. In addition, some data based on graphical figures or other formats (median, interquartile range) were not estimated in our study for the unwanted error.

### Data Extraction

A standardized Excel spreadsheet was prepared for data extraction. Data from the included studies were extracted and summarized independently by two independent authors according to the inclusion and exclusion criteria. The information from each study was extracted as follows: author, publication year, country, diagnostic criteria of acute pancreatitis, source of acute pancreatitis patients, detection method for resistin, sample type, age and gender of samples, sample size, the levels of resistin (mean, standard deviation, or *P-*value). When discrepancies occurred during in the process of data extraction, a third person was consulted to resolve these issues. Finally, data were rechecked by an independent author for accuracy.

### Statistical Analysis

Pre-extracted means and standard deviations or sample size and *P-*values were employed to calculate Hedges' g statistic. Hedges' g and its 95% CI were selected to evaluate the significant difference of peripheral resistin levels in severe and mild acute pancreatitis. Statistical heterogeneity test across studies was carried out using the I^2^ value and a chi-square-based Q-test. When obvious heterogeneity existed, a random-effect model was selected to calculate the corresponding effect size; otherwise, a fixed-effect model was selected. To verify the effect of a single study on the comprehensive results directly, we conducted sensitivity analyses for statistically significant ES estimates by removing each study from the analysis in turn. A funnel plot was drawn to test the publication bias. When the *P-*value < 0.05, it was considered statistically significantly different in the present study.

To explore the source of heterogeneity, subgroup analyses for sample type and ethnicity based on available information. A Galbraith plot was drawn to identify potential studies affecting heterogeneity across studies. Unrestricted maximum-likelihood random-effects meta-regressions of effect sizes were performed to verify whether some theoretical covariates (mean age, gender (male proportion) of samples, sample size, and publication year) serve as confounders that affected our results. We conducted all statistical analyses using Comprehensive Meta-Analysis version 3 software (version 3; Biostat Inc) and STATA15 software (StataCorp, College Station, TX, USA).

## Results

### Literature Search Results

**Figure 1:**
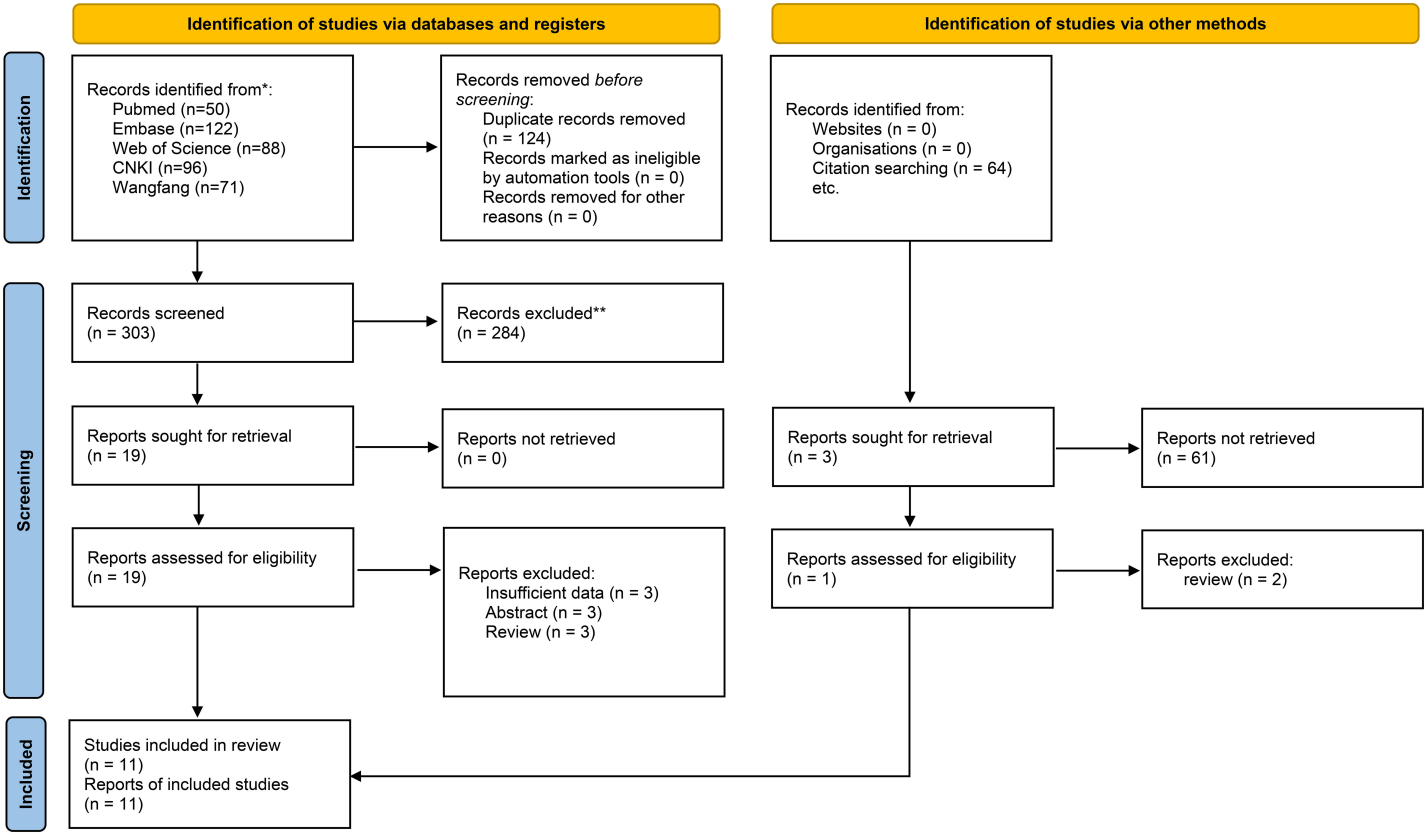
Flow diagram of study identification.

There were 428 records identified by electronic search with the retrieval key search terms. A total of 50 records were retrieved from PubMed, 122 records from Embase, 88 records from Web of Science, 96 records from CNKI, 71 records from Wan-fang database, and one additional record identified through other sources. After removing 124 duplications and 284 unrelated records based on review of titles or abstracts, twenty records were further screened by full-text reading. Three records without sufficient data were excluded. Three records were meeting abstracts, and three records were reviews. Therefore, eleven records (11 studies) with 892 acute pancreatitis patients were included in the present meta-analysis to evaluate the association between the levels of resistin and the severity of AP (11, 22–31). The flow diagram is presented in Figure 1.

### Study Characteristics

Of the eleven included studies, five studies were carried out in China, two studies in Turkey, one study in India, one study in Germany, one study in Lithuania, and one in Saudi Arabia. All of the included studies were published after 2010. We noted that serum resistin concentration was detected in nine studies and plasma resistin concentration was tested in two. The levels of peripheral resistin were determined using ELISA or radioimmunoassay kit. The minimum and maximum mean ages of the samples of the included studies were 39.16 and 60.38, respectively. The levels of peripheral resistin were detected in patients with acute pancreatitis within 24 h of admission in nine studies (11, 22–25, 27–30). The characteristics of the included studies are presented in Table 1.

**Table 1:** Characteristics of the studies included in the meta-analysis.

**Sample**	**First author**	**Country**	**Diagnostic**	**Source**	**Detection method**	**Sample size**				**Severe AP**		**Mild AP**		
	**Publication year**		**Criteria**			**Severe AP**	**Mild AP**	**Mean age (years)**	**Gender (M/F**	**Mean ±SD**	**Unit**	**Mean ±SD**	**Unit**	**P**
Serum	Singh et al. (26)	India	Revised Atlanta criteria	A tertiary care center in North India	ELISA	77	53	39.16	100/30	1.24 ± 1.72	ng/ml	1.39 ± 2.45	ng/ml	NA
Plasma	Kibar et al. (11)	Turkey	Atlanta classification	The Gastroenterology Clinic, Erzurum Education and Research Hospital, Turkey	ELISA	22	37	60.38	21/38	28.90 ± 5.22	ng/ml	18.30 ± 6.95	ng/ml	0.01
Serum	Kisaoglu et al. (25)	Turkey	Atlanta classification	The Department of General Surgery, Ataturk University Medical School	ELISA	17	17	59.12	24-Oct	26.48 ± 12.03	ng/dl	23.50 ± 12.3	ng/dl	0.492
Serum	Schäffler et al. (22)	Germany	Clinical, laboratory, and radiological findings	The University Hospital of Regensburg, Germany	ELISA	41	9	57.9	30/20	74.14 ± 94.86	ng/ml	35.90 ± 54.6	ng/ml	0.04
Serum	Zhu et al. (27)	China	Guidelines for diagnosis and treatment of acute pancreatitis in China	The Second People's Hospital Of Yunnan Province	Radioimmunoassay kit	48	72	46.68	67/53	59.4 ± 6.74	μg/L	27.18 ± 3.82	μg/L	NA
Plasma	Wang et al. (28)	China	Guidelines for diagnosis and treatment of acute pancreatitis in China	Fenyang Hospital, Shanxi Province	Radioimmunoassay kit	57	60	42.57	64/53	59.38 ± 6.72	μg/L	27.15 ± 3.84	μg/L	NA
Serum	Yuan et al. (29)	China	Guidelines for diagnosis and treatment of acute pancreatitis in China	Ganyu District People's Hospital of Lianyungang City	ELISA	17	31	48.93	29/19	6.73 ± 2.46	mg/L	2.68 ± 1.07	mg/L	NA
Serum	Chen et al. (30)	China	Guidelines for diagnosis and treatment of acute pancreatitis in China	The First Affiliated Hospital of Hainan Medical College	ELISA	23	31	44.8	32/22	143.85 ± 61.23	μg/L	42.07 ± 11.59	μg/L	NA
Serum	Li et al. (31)	China	Guidelines for diagnosis and treatment of acute pancreatitis in China	Jilin Province People's Hospital	ELISA	14	62	40.93	43/33	23.40 ± 1.78	ng/ml	14.20 ± 3.43	ng/ml	NA
Serum	Karpavicius et al. (24)	Lithuania	Revised Atlanta criteria	Four Lithuanian hospitals	ELISA	20	82	55.72	50/52	20.20(31.75)^a^	ng/ml	10.70(8.65)^a^	ng/ml	<0.001
Serum	Al-Maramhy et al. (23)	Saudi Arabia	Atlanta classification	King Fahd Hospital, Saudi Arabia	ELISA	22	80	45	42/60	17.5	ng/ml	16.82	ng/ml	0.188

a *Data presented as median (IQR); ELISA, Enzyme Linked Immunosorbent Assay; NA, not available*.

### Quantitative Data Synthesis

**Figure 2:**
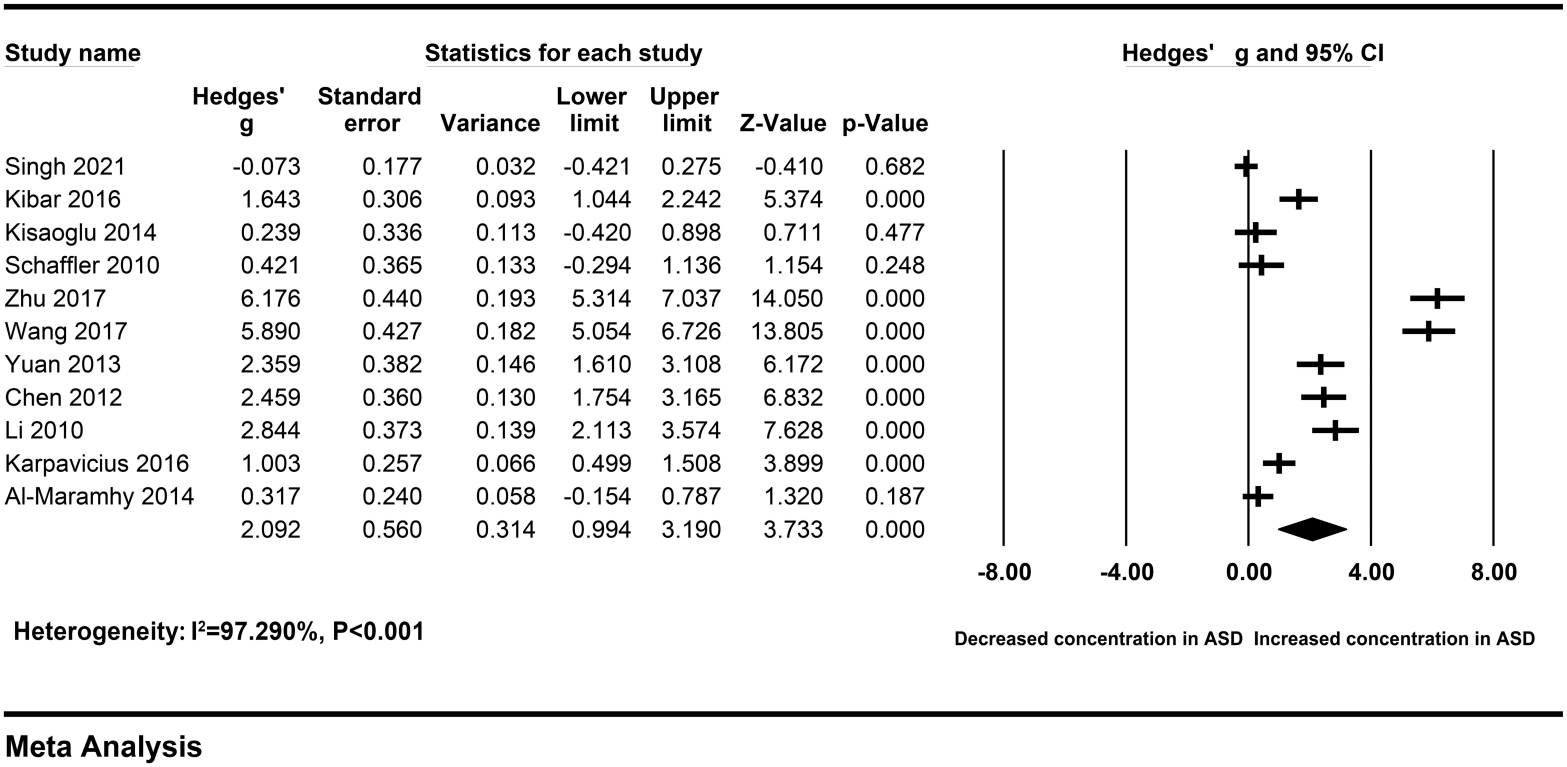
Forest plot for the random-effect meta-analysis.

The pooled Hedges' g and its corresponding 95% CI were calculated based on the extracted data of eleven studies compassing 358 patients with severe AP and 534 patients with mild AP. Obvious heterogeneity across studies were found in main analysis (I^2^ = 97.290%, *P* < 0.001). Therefore, a random-effect model was used to calculate the pooled ES and its corresponding 95% CI. The results of the overall comparison revealed that, peripheral resistin levels were statistically elevated in patients with severe AP compared with patients with mild AP (Hedges' g = 2.092, 95% CI: 0.994–3.190, *P* < 0.001) (Figure 2) (Table 2).

**Table 2:** Summary of meta-analysis results.

				**Tests of association**				**Tests of heterogeneity**		
**Groups**	**Studies (*n*)**	**Case (*n*)**	**Control (*n*)**	**Model**	**Hedges'g [95%CI]**	**Z**	**P–value**	**Q-value**	**P-value**	**I^**2**^ (%)**
Total	11	358	534	RE	2.092 [0.994–3.190]	3.733	<0.001	368.952	<0.001	97.290
Subgroups										
Serum	9	279	437	RE	1.723 [0.644–2.803]	3.128	0.002	244.353	<0.001	96.726
Chinese	5	159	256	RE	3.933 [2.318–5.549]	4.772	<0.001	87.537	<0.001	95.430
After removing studies	4	76	161	FE	2.254 [1.910–2.598]	12.843	<0.001	6.902	0.075	56.533

*RE, random-effect model; FE, fixed-effect model*.

### Heterogeneity Analysis

**Figure 3:**
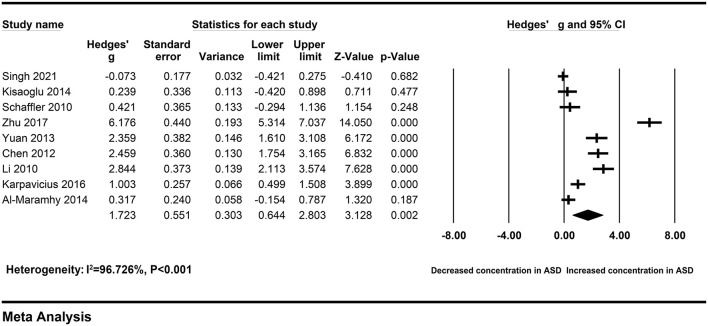
Forest plot for the random-effect meta-analysis of the serum subgroup.

**Figure 4:**
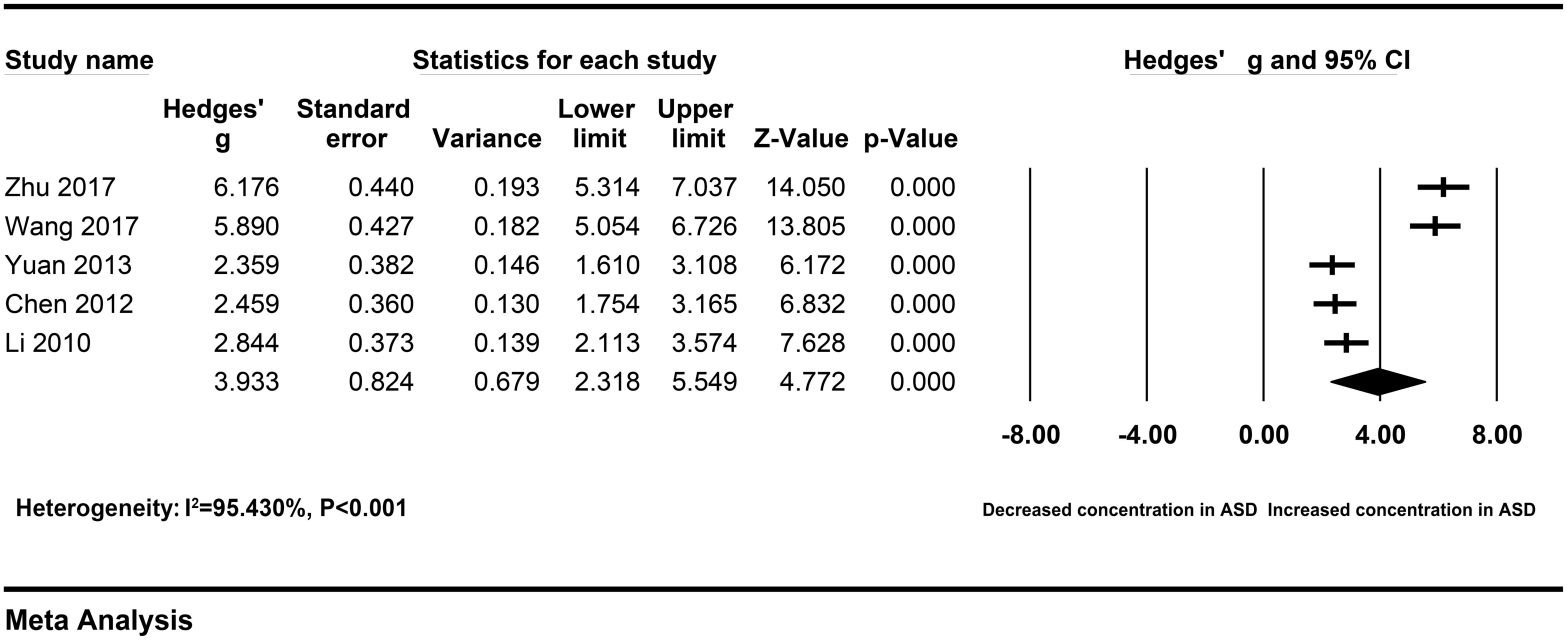
Forest plot for the random-effect meta-analysis of the Chinese subgroup.

To explore the sources of heterogeneity across studies, subgroup analyses were according to different sample types and ethnicity. The correlation between elevated peripheral resistin levels and severity of AP did not change obviously in the subgroup with the serum samples (Hedges' g = 1.723, 95% CI: 0.644–2.803, *P* = 0.002) and Chinese population (Hedges' g = 3.933, 95% CI: 2.318–5.549, *P* < 0.001) (Figures 3, 4). However, heterogeneity across studies did not decrease significantly in serum (I^2^ = 96.726%, *P* < 0.001) or Chinese (I^2^ = 95.430%, *P* < 0.001) subgroups. It suggested that sample types and ethnicity did not address the source of heterogeneity across studies.

**Figure 5:**
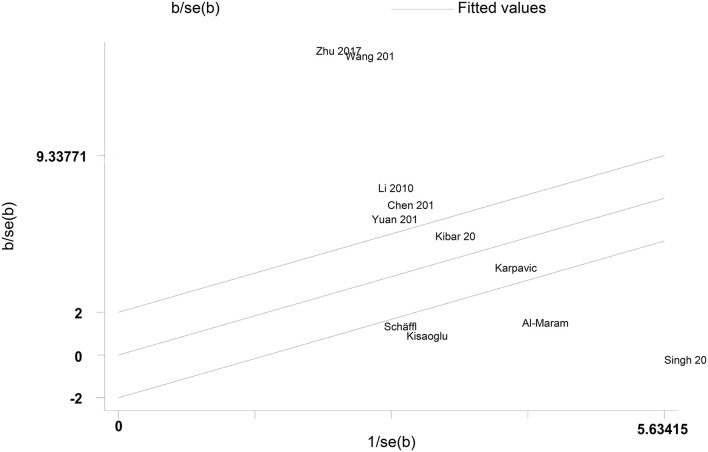
Galbraith plot for the random-effect meta-analysis.

**Figure 6:**
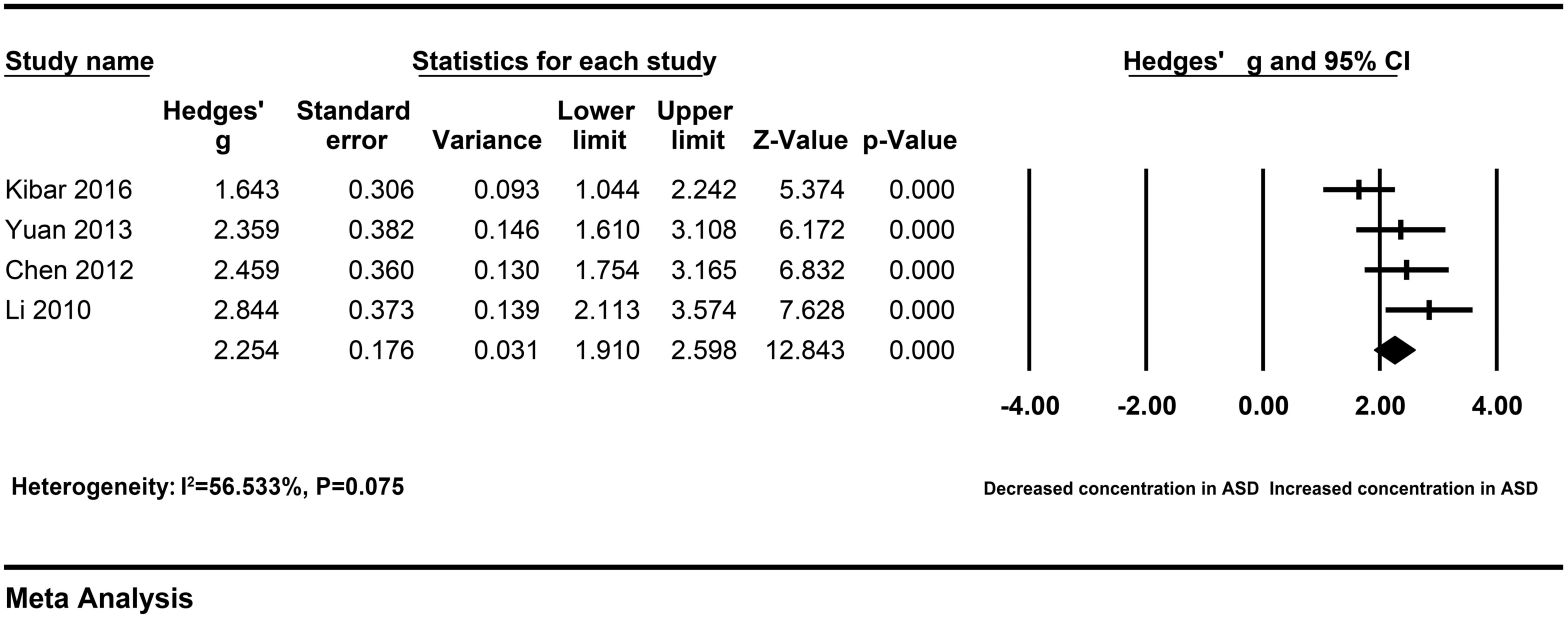
Forest plot for the fixed-effect meta-analysis after removing the studies outside the boundaries of the Galbraith plot.

A Galbraith plot was drawn according to the extracted data of included studies to identify the studies deviated from baseline, which may affect heterogeneity across studies. As shown in Figure 5, seven studies (22–28) were labeled for displacing from the center of the zero line in the Galbraith plot. After removing these studies, heterogeneity across studies decreased obviously (I^2^ = 56.533%, *P* = 0.075). Moreover, peripheral resistin levels were still significantly higher in patients with severe AP than patients with mild AP (Hedges' g = 2.254, 95% CI: 1.910–2.598, *P* < 0.001) (Figure 6) (Table 2).

**Figure 7:**
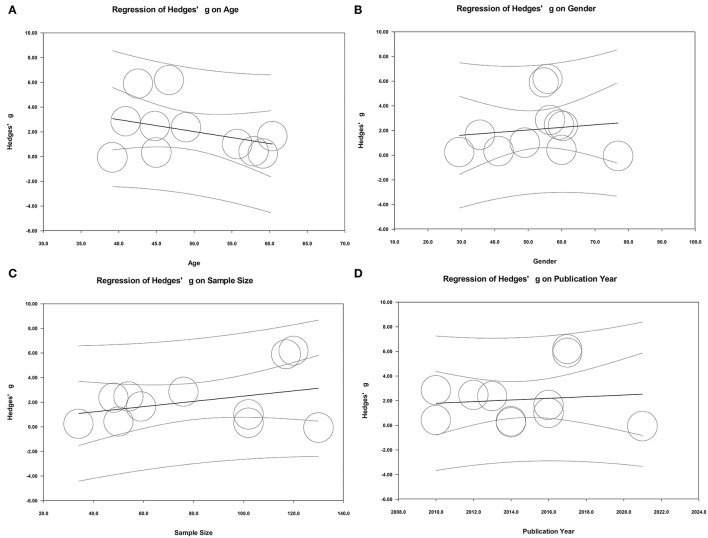
**(A–D)** Meta-regression in all studies.

In addition to the above relevant categorical variables (sample type and ethnicity) and potential studies affecting heterogeneity, the effects of some potential continuous variables (age, gender, and sample size) on heterogeneity were also evaluated by performing meta-regression analyses. The results of meta-regression suggested that age, gender of samples, sample size, and publication year did not play important roles in moderating effects on the present results (Figure 7).

### Sensitivity Analysis

**Figure 8:**
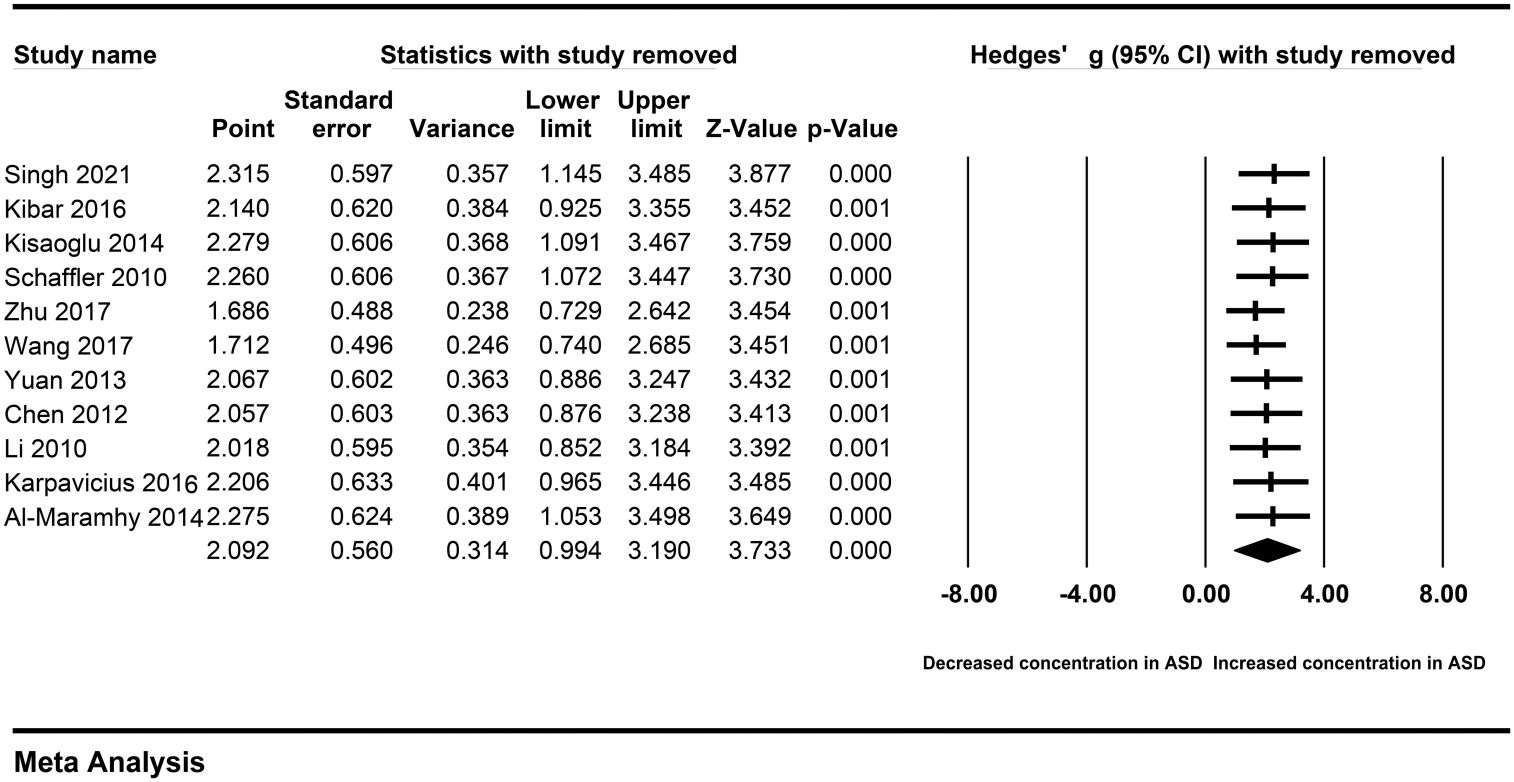
Sensitivity analysis.

A single study may affect the overall result, and cause obvious heterogeneity across studies. Therefore, we performed the sensitivity analysis by removing each study sequentially. The association between peripheral resistin and the severity of AP was also observed after removing a single study sequentially (Figure 8). Similarly, we did not found heterogeneity across studies decreased significantly. Therefore, a single study did not affect our results.

### Publication Bias

**Figure 9:**
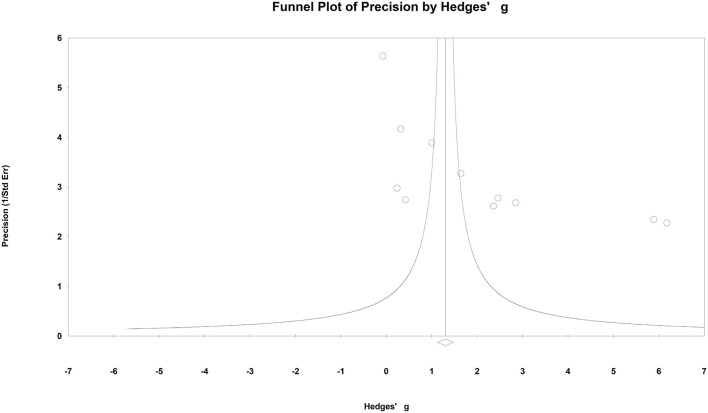
Funnel plot of precision using Hedges' g statistics.

A funnel plot was presented in Figure 9. No obvious publication bias was found in this meta-analysis.

## Discussion

So far as we know, this is the first meta-analysis to investigate the relation of peripheral resistin levels to severity of acute pancreatitis. The main finding of our meta-analysis was that there was a significant association between peripheral levels of resitin and severity of acute pancreatitis. There was no single study affecting the overall outcomes, which was verified by performing a sensitivity analysis. The funnel plot showed that no obvious publication bias in our study. Resistin has been implicated to play important roles in the development of insulin resistance and affecting pancreatic function (20). Despite the disagreements in the effects of peripheral resistin on severity of AP, our present study combining the published data suggested that peripheral levels of resistin were significantly higher in patients with severe AP compared with patients with mild AP, which may provide us some clues about the marker for the accurate early prediction of the severity of acute pancreatitis. We noticed that the levels of peripheral resistin were detected in patients with acute pancreatitis within 24 h of admission in most of the included studies. Early screening the levels of resistin in peripheral blood of patients with acute pancreatitis after admission may help clinicians better master the patient's condition and assess the risk of severe acute pancreatitis in patients. In addition, further developing effective interventions after early prediction of the severity of acute pancreatitis may enhance the prognosis of acute pancreatitis.

It has not escaped our notice that there was obvious heterogeneity across studies in both overall analysis and subgroup analysis. Although subgroup analyses with limited information did not define the source of heterogeneity in our study, peripheral resistin levels remained significantly higher in both serum and Chinese subgroups. A Galbraith plot was drawn to identify the source of heterogeneity in the included studies. Seven studies were removing from the overall analysis and heterogeneity across studies was statistically decreased. These studies may cause obvious heterogeneity. In addition, meta-regression analyses to adjust for potential moderators were performed to explore the source of heterogeneity. The results of meta-regression indicated that age and gender of samples, sample size and publication year did not play an important role in moderating the outcomes of the meta-analysis. These factors may be not the potential confounding factor. More information was needed to identify the potential source of heterogeneity.

What is the role of the elevated resistin levels on the severity of AP? Oxidative stress and inflammatory response are regarded as major causative factors in acute pancreatitis. Peripheral resistin was reported enhancing environmental factors-induced IL-6 and ROS expression in pancreatic acinar cells, which resulted in endothelial cells injury and the release of endothelin-1 (21, 32). Endothelin-1 plays an important role in the pathogenesis of pancreatitis by a decrease in pancreatic perfusion and contribute to oxidative stress and inflammatory response (33). Thus, there seems to be a causal link between peripheral resistin and the severity of AP. The exact mechanism of peripheral resistin affecting the severity of AP should be further verified by well-designed animal studies.

Some inherent limitations should be pointed out in our meta-analysis. First, the sample size was moderate, especially in some subgroups, and lots of information was unavailable. In terms of included studies, substantial variability in sampling methods, sample sizes, detecting methods, and diagnostic criteria should be further assessed despite of some factors detected in the study. We should realize that obvious heterogeneity still exists. The source of heterogeneity may be further explored with more detailed information. Second, the numbers of studies that analyzed plasma resistin levels from patients with AP were small. Whether the association between resistin levels and acute pancreatitis was applicable in plasma should be further verified with more well-designed studies. Third, despite of elevated resistin levels in patients with severe AP, whether an increase in resistin levels is a cause for onset and exacerbation of acute pancreatitis or an epiphenomenon remains unclear. We should recognize the clinical significance of elevated peripheral resistin levels dialectically. More basic studies were needed to confirm the causal relationship of resistin and acute pancreatitis. Four, more longitudinal studies on resistin levels and acute pancreatitis should be performed to explore the development of acute pancreatitis, instead of cross-sectional studies.

## Conclusions

Our meta-analysis suggested that increased resistin levels in peripheral blood may be a hallmark of severe acute pancreatitis, which may provide us some new insights about the early diagnosis. Well-designed studies are needed to verify the mechanism of peripheral resistin on the severity of AP.

## Data Availability Statement

The raw data supporting the conclusions of this article will be made available by the authors, without undue reservation.

## Author Contributions

JY, ML, and JG were the main researchers in this study and took part in the study conceptualization, literature review, data extraction, analysis, and writing of the manuscript. SW and YG revised the manuscript editing. JG, YT, and XC planned the study and revised the manuscript. All authors read and approved the final manuscript.

## Conflict of Interest

The authors declare that the research was conducted in the absence of any commercial or financial relationships that could be construed as a potential conflict of interest.

## Publisher's Note

All claims expressed in this article are solely those of the authors and do not necessarily represent those of their affiliated organizations, or those of the publisher, the editors and the reviewers. Any product that may be evaluated in this article, or claim that may be made by its manufacturer, is not guaranteed or endorsed by the publisher.

## Abbreviations

AP: acute pancreatitis
SAP: severe acute pancreatitis
CNKI: China National Knowledge Infrastructure
ES: effect size
CI: confidence interval.
